# The Gut-Bone Axis and Skeletal Health: Regulatory Mechanisms and Therapeutic Applications of Plant-Derived Bioactive Compounds

**DOI:** 10.3390/biom16060912

**Published:** 2026-06-19

**Authors:** Tianzhu Zhang, Yufei Li, Jiahui Pei, Qingxia Zhang, Fengyun Lin, Shuzhen Li

**Affiliations:** 1 College of Pharmacy, Chongqing Medical and Pharmaceutical College, Chongqing 401331, China; 10776@cqmpc.edu.cn; 2 Engineering Research Center for Digital and Intelligent Biomedical Equipment and Novel Dosage Forms, Chongqing Medical and Pharmaceutical College, Chongqing 401331, China; 3 Department of Food Science, College of Public Health, Shenyang Medical College, Shenyang 110034, China; liyufei030099@163.com (Y.L.); pjh9249808@163.com (J.P.); 18363342407@163.com (Q.Z.); 4 Department of Immunology, College of Basic Medical Sciences, Shenyang Medical College, Shenyang 110034, China

**Keywords:** bone health, dietary intervention, gut–bone axis, phytochemicals, plant-derived bioactive compounds

## Abstract

The gut microbiota and its metabolites, as components of the gut–bone axis, play a pivotal role in regulating skeletal homeostasis through the bidirectional communication network. In this systematic review, evidence was collected from mainstream databases following standardized inclusion/exclusion criteria for screening, to comprehensively retrieve and screen eligible studies from multiple mainstream databases according to standardized inclusion and exclusion criteria, and systematically summarize current research progress on plant-derived bioactive compounds targeting the gut–bone axis for skeletal health regulation. This review systematically explores the underlying mechanisms of the gut–bone axis and critically evaluates the regulatory effects and therapeutic potential of plant-derived bioactive compounds. Particular attention is given to targeted interventions involving prebiotics, probiotics, synbiotics, and plant-rich diets or functional foods. Among these interventions, synbiotics represent the most successful strategy and show the most prominent therapeutic possibilities in bone-related disorders. Different from single prebiotics (only nourish endogenous intestinal microbes), individual probiotics (easy to be degraded in gastrointestinal tract with poor colonization) and ordinary plant-rich diets (unfixed effective dosage and weak targeting property), synbiotics combine prebiotic carriers and viable probiotic strains to produce complementary advantages, which is the core reason for its outstanding therapeutic prospect against bone diseases. Synbiotics exert synergistic effects on gut microecology, mineral absorption, and immune regulation, leading to more robust and consistent improvements in bone health than single prebiotics, probiotics, or general plant-rich diets. They have been verified in preclinical and clinical studies to ameliorate osteoporosis and related skeletal diseases via the gut–bone axis. These strategies offer novel insights into the prevention and treatment of bone metabolic disorders, such as osteoporosis, by targeting the gut–bone axis with phytochemicals. Key outcomes of this review include that synbiotics, soy isoflavones, naringin, curcumin, and resveratrol effectively improve bone mineral density, restore gut microbiota balance, and inhibit pathological bone resorption via the gut–bone axis. Collectively, the above bioactive substances realize bone protection mainly by reshaping gut flora, elevating mineral uptake and suppressing excessive osteoclast activity. Representative cases include soy isoflavones mitigating estrogen-deficient bone loss in OVX models, naringin improving the trabecular microarchitecture, and probiotic *BL*-11 promoting longitudinal bone growth in children. Future directions will focus on clarifying dose–response relationships, developing standardized synbiotic formulations, constructing microbiome-guided precision diets, and conducting large-sample randomized controlled trials to translate plant-derived compounds into clinical therapies.

## 1. Introduction

The gut–bone axis refers to the functional connection between the gut and the skeletal system, established through a complex bidirectional communication network. In recent years, this concept has become a frontier area in the research of metabolic bone diseases [1]. The gut microbiota, as the largest symbiotic ecosystem in the human body, participates in the maintenance of bone metabolic homeostasis through multiple pathways, including immune regulation, nutrient absorption, endocrine signaling, and the production of microbial metabolites. Under normal conditions, the gut microbiota maintains a relatively stable microecological balance. However, imbalance (i.e., dysbiosis) induced by antimicrobials, poor diet, aging and miscellaneous external factors disrupts bone metabolism via gut–bone axis signaling pathways, eventually promoting the onset of osteoporosis and other metabolic skeletal disorders [2].

In addition to plant-derived bioactive components, remarkable biotechnological advances have been achieved for bone-related disorders, including stem cell transplantation, gene editing therapy, recombinant protein drugs, and bone tissue engineering [3,4]. Mesenchymal stem cell transplantation can promote osteogenic differentiation and repair bone defects [5]; CRISPR/*Cas9*-mediated gene editing enables targeted regulation of genes related to osteoclast activation and bone formation [6]; recombinant osteogenic proteins and growth factors have been used to accelerate bone regeneration [7]; and bone tissue engineering provides personalized scaffolds for bone defect repair [8]. These biotechnologies provide new strategies for the treatment of severe bone injury and metabolic bone diseases.

Plant-based proteins constitute an important component of the human diet, primarily serving as the source of essential amino acids. In addition, plants are rich in bioactive phytochemicals, such as polyphenols, plant-derived amino acids, and peptides [9]. In recent years, substantial progress has been made in preclinical studies, clinical trials, and commercial translation of plant-derived bioactive components for skeletal health. Soy isoflavones, curcumin, resveratrol, and naringin have been widely validated in OVX animal models to attenuate bone loss, improve bone microarchitecture, and regulate bone turnover markers [10,11,12,13]. Multiple randomized controlled trials have confirmed that soy isoflavones (especially genistein) and equol-producing strains effectively maintain bone mineral density and reduce bone resorption in postmenopausal women [10,14]. These recent advances strongly support the clinical value and translational potential of plant-based constituents in targeting the gut–bone axis for skeletal health. These compounds exhibit significant potential in the prevention and treatment of osteoporosis via the gut–bone axis, primarily by modulating the composition and function of the gut microbiota [15,16,17]. Studies have shown that the incidence of osteoporotic fractures is lower in Asian populations than in Western populations, which may be related to the high plant-based food intake in traditional Asian diets. Traditional Asian diets are rich in dietary fiber, phytochemicals, and soy products, which can regulate gut microbiota and promote bone health. Western diets usually contain more fat and refined carbohydrates, which are less favorable to bone metabolism [18]. Currently, large-scale direct comparative statistics are still insufficient, but existing evidence supports that Asian dietary patterns are more beneficial to skeletal health.

In summary, the gut–bone axis serves as a critical bridge linking gut microbiota, plant-derived bioactive compounds, and skeletal homeostasis. Despite substantial progress, the field still faces several bottlenecks, including unclear strain-specific and compound-specific mechanisms, insufficient large-scale clinical evidence, low bioavailability of phytochemicals, and lack of standardized intervention regimens. To address these challenges, emerging strategies such as novel delivery systems, synbiotic combinations, microbiome-guided precision diets, and multi-omics validation have shown promising potential. This comprehensive review systematically summarizes the regulatory mechanisms of the gut–bone axis, evaluates the effects of plant-derived components, discusses targeted intervention strategies, and highlights translational challenges and future directions. By integrating mechanistic insights, preclinical evidence, clinical advances, and translational prospects, this review aims to enrich the current understanding of the gut–bone axis and provide a theoretical foundation and practical guidance for subsequent research and clinical development of plant-based therapies against osteoporosis and other metabolic bone diseases. A systematic literature search was performed for publications from 2016 to 2026, focusing on the gut–bone axis, plant-derived bioactive compounds, and skeletal health. The electronic databases included PubMed, Web of Science, ScienceDirect, and CNKI. Search terms were a combination of “gut–bone axis”, “gut microbiota”, “plant-derived bioactive compounds”, “phytochemicals”, “osteoporosis”, “bone metabolism”, and “skeletal health”. Only peer-reviewed original articles, clinical trials, and systematic reviews published in English or Chinese were considered. Exclusion criteria comprised conference abstracts, letters, reviews without original data, duplicate publications, and studies with incomplete outcome data. Titles and abstracts were first screened independently by two authors, followed by full-text evaluation for eligibility. Reference lists of included articles were manually checked to identify additional relevant studies.

## 2. Mechanistic Role of the Gut–Bone Axis

### 2.1. Immunoregulatory Mechanisms

**Figure 1:**
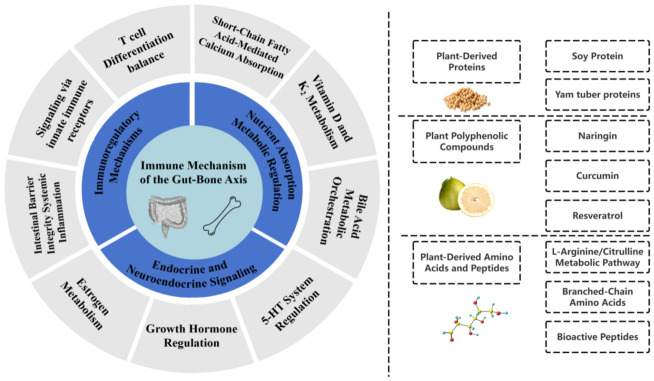
Immunological mechanisms of the gut–bone axis (GBA) and modulatory effects of plant-derived compounds on the gut–bone axis.

The gut microbiota influences bone metabolic homeostasis by modulating the host immune system. This process primarily involves the following three major immunological pathways, summarized in Figure 1.

#### 2.1.1. T-Cell Differentiation Balance

The gut microbiota directly regulates the Th17/Treg cell balance. IL-17 secreted by Th17 cells can stimulate osteoblasts to express RANKL, thereby promoting osteoclast differentiation and bone resorption [19]. In contrast, regulatory T (Treg) cells inhibit bone resorption by producing interleukin-10 (IL-10, a kind of anti-inflammatory cytokine) and transforming growth factor-beta (TGF-β) [20]. Research has demonstrated that specific probiotic strains, including *Bacillus clausii* and *Lactobacillus* species, significantly reduce ovariectomy (OVX)-induced bone loss in mice by modulating the Th17/Treg cell balance [21,22]. Moreover, in a postmenopausal osteoporosis model, intervention with soybean isoflavones significantly reduced the levels of tumor necrosis factor-alpha (TNF-α) in serum, suppressed activation of osteoclast precursor cells (CD4^+^/GR1^−^) in bone marrow, and mitigated estrogen deficiency-induced inflammatory bone loss [23]. Given that pro-inflammatory cytokines such as TNF-α enhance Th17 cell differentiation and function while potentially suppressing the stability of Treg cell; these collective effects suggest that soybean isoflavones may modulate the Th17/Treg cell balance via regulation of inflammatory signaling pathways. This proposed mechanism likely contributes to their bone-protective effects [24].

#### 2.1.2. Signaling via Innate Immune Receptors

As pattern recognition receptors (PRRs), Toll-like receptors (TLRs) and NOD-like receptors (NLRs) mediate osteoimmunological regulation by detecting microbe-associated molecular patterns (MAMPs) [25]. For instance, TLR5 engagement can upregulate RANKL production in osteolineage cells by flagellin triggers NF-κB signaling. This enhances osteoclast precursor differentiation, driving pathological bone resorption [26]. In contrast to TLRs, NOD1/NOD2 receptors sense peptidoglycan fragments to modulate cortical bone homeostasis. Impaired osteoclastogenesis in NOD2 marrow macrophages provides genetic evidence that NOD2 signaling is essential for bacteria-triggered bone loss, revealing a targetable axis in infective osteolysis [27].

#### 2.1.3. Intestinal Barrier Integrity and Systemic Inflammation

Disruption of gut microbial homeostasis impairs intestinal barrier integrity, facilitating bacterial and endotoxin translocation. This cascade propagates systemic inflammation through PRR activation (e.g., TLR4/NOD2), ultimately driving osteoclastogenesis and bone loss [28]. Notably, blooms in proteobacteria, a signature of dysbiosis, impair gut barrier integrity through suppression of tight junction proteins (like claudin-1 and occluding). This permits systemic leakage of LPS, a TLR4 agonist that polarizes macrophages toward a pro-osteoclastogenic phenotype via IL-1β/IL-6/TNF-α secretion. Consequently, enhanced osteoclast differentiation drives pathological bone resorption, mechanistically coupling enteric dysbiosis to skeletal deterioration [29]. In a study on metronidazole-induced bone loss, pathologic overgrowth of the conditional pathogen *Klebsiella variicola* and its pro-inflammatory extracellular vesicles (EVs) were shown to infiltrate bone tissue. These EVs directly activated inflammatory responses in the bone marrow niche and promoted osteoclast differentiation, driving osteolytic pathology [30].

### 2.2. Nutrient Absorption and Metabolic Regulation

Gut microbiota orchestrate mineral bioavailability and modulate core regulators of bone metabolism through the following immunomodulatory and endocrine pathways.

#### 2.2.1. Short-Chain Fatty Acid-Mediated Calcium Absorption

Gut microbiota ferment dietary fiber, which generates short-chain fatty acids (SCFAs; e.g., acetate, propionate and butyrate are the major representative SCFAs capable of mediating calcium absorption), and enhance intestinal calcium absorption through three synergistic mechanisms: (1) luminal acidification via proton release; (2) stimulation of enterocyte proliferation; (3) upregulation of calcium transporter expression (notably TRPV6 and CaT1) [31]. SCFAs can enhance calcium absorption and regulate bone metabolism by acidifying the intestinal lumen. This pH value reduction solubilizes ionized calcium, preventing its precipitation with phosphate, and thereby increasing its bioavailability for skeletal mineralization [32]. Treatment of mice under germ-free (GF) conditions resulted in reduced expression of pro-inflammatory cytokines TNF-α and IL-6, as well as decreased numbers of CD4^+^ T cells and osteoclasts, compared with specific pathogen-free (SPF) controls. This attenuated immuno-inflammatory status correlates with superior bone mass accrual, indicating that microbiota-derived SCFAs regulate osteoclastogenesis through immunomodulation and suppression of local inflammatory reactions [33]. Furthermore, SCFAs directly enhance bone anabolism by inhibiting histone deacetylases (HDACs), modulating chromatin accessibility and transcriptional activation of osteogenic gene networks in osteoblasts [34,35].

#### 2.2.2. Vitamin D and K_2_ Metabolism

Vitamin D enhances intestinal calcium absorption through genomic activation of epithelial calcium channels. Crucially, gut microbiota composition modulates the bioavailability of active vitamin D metabolites by regulating intestinal absorption efficiency, hepatic and renal metabolic activation, and enterohepatic circulation of vitamin D precursors, thereby affecting the circulating level of biologically active 1,25(OH)_2_D_3_ [36]. Clinically, *Lactobacillus rhamnosus* supplementation elevates serum 25-hydroxyvitamin D3 levels, indicating microbial regulation of vitamin D endocrinology [37]. Vitamin K_2_, as an essential cofactor for the γ-carboxylation of osteocalcin, acts a critical post-translational modification conferring mineralization competence. Notably, the biosynthesis of active vitamin K_2_ was mediated by specific commensal gut microbiota, such as *Bifidobacterium longum* [38]. Vitamin K_2_ can accelerate fracture healing and osteogenesis through extrahepatic tissue distribution following gut microbial synthesis. Upon activation of osteocalcin (OCN), vitamin K_2_ suppresses NF-κB signaling to modulate the expression of matrix Gla protein (MGP) and the level of calcification inhibitors in vasculature and cartilage. This cascade concurrently can inhibit osteoclastogenesis while promoting osteoblast (OB) differentiation [39].

#### 2.2.3. Bile Acid Metabolic Orchestration

Primary bile acids undergo gut microbiota-mediated biotransformation into secondary bile acids, acting the role of signaling agonists for both the nuclear receptor farnesoid X receptor (FXR) and the G protein-coupled bile acid receptor TGR5 [40]. FXR signaling suppresses osteoclastogenesis by enhancing transcriptional activation of osteoprotegerin (OPG) in osteoblasts, thereby blocking RANKL-mediated osteoclast differentiation [41]. TGR5 activation suppresses osteoclast differentiation from macrophage precursors via cAMP/PKA signaling. Concurrently, bioactive components in plant proteins (such as soy isoflavones and bioactive peptides) optimize bile acid metabolic profiles, which indirectly modulates bone remodeling processes through complementary pathways.

### 2.3. Endocrine and Neuroendocrine Signaling

The gut microbiota regulates bone metabolism partially through modulation of pivotal endocrine and neuroendocrine pathways. Key mechanisms include modification of estrogen metabolism, the impact on the growth hormone-insulin-like growth factor 1 (GH-IGF-1) axis, and regulation of the serotonin (5-hydroxytryptamine, 5-HT) system.

#### 2.3.1. Estrogen Metabolism

Microbial β-glucuronidases facilitate enterohepatic estrogen recirculation by liberating bioactive estrogens from glucuronide conjugates. Soy isoflavones are metabolized by gut microbiota into equol, a potent estrogenic metabolite, that inhibits bone resorption via estrogen receptor agonism. In ovariectomized (OVX) mice, soy isoflavone intervention significantly elevated the level of serum estradiol (E2) while reducing the expression of serum C-terminal telopeptide type I collagen (CTX-1) (a bone resorption marker), demonstrating efficacy comparable to the anti-osteoporotic drug denosumab [42].

#### 2.3.2. Growth Hormone Regulation

Specific probiotics modulate growth hormone (GH) secretion. Bifidobacterium animalis subsp. 188 lactis *BL*-11, a high-yield producer of γ-aminobutyric acid (GABA), improves sleep architecture to potentiate pulsatile GH release. GH stimulates hepatic insulin-like growth factor 1 (IGF-1) production, which directly enhances osteoblast proliferation and differentiation. In a randomized controlled trial, children receiving *BL*-11 intervention exhibited a mean height increase of 2.6 cm over 12 weeks, significantly greater than placebo controls [43].

#### 2.3.3. Serotonin (5-HT) System Regulation

The gastrointestinal tract accounts for >90% of systemic serotonin (5-hydroxytryptamine, 5-HT) synthesis. Gut microbiota critically regulate 5-HT biosynthesis through modulation of tryptophan metabolic flux, primarily by controlling precursor bioavailability and tryptophan hydroxylase 1 (TPH1) activity in enterochromaffin cells, and related clinical trials further confirm that probiotic intervention can remodel gut microbiota and effectively regulate peripheral 5-HT levels in human subjects [44]. Circulating serotonin significantly inhibits osteoblast proliferation by downregulating the expression of cyclinD1 through HTR1B receptors. Vegetarian dietary regimens have shown promise in attenuating such osteogenic suppression by enriching SCFA-producing microbiota. These microbiota reduce intestinal 5-HT biosynthesis through TPH1 inhibition, thereby alleviating 5-HT-mediated inhibition of bone formation [45].

## 3. Regulatory Effects of Plant Proteins and Bioactive Components on the GBA

The regulatory effects of numerous plant-derived bioactive components on the GBA have garnered increasing scientific attention, and are regarded as a promising strategy for maintaining skeletal homeostasis [46] (Figure 1 and Table 1). Current evidence showed a focused examination of three core plant-derived components, namely proteins and their bioactive derivatives (e.g., soy protein and Indian yam *Dioscorea oppositifolia* protein), polyphenolic compounds (e.g., naringin, curcumin, and resveratrol), and amino acids/peptides (including L-arginine/citrulline, branched-chain amino acids, and specific bioactive peptides).

### 3.1. Plant-Derived Proteins

#### 3.1.1. Soy Protein

Soy protein represents the most extensively characterized category of plant-derived proteins modulating bone metabolism via the GBA, primarily due to its core bioactive constituents, soy isoflavones (SIs). SIs contain three kinds of principal aglycones: genistein, daidzein, and glycitein respectively [47]. In osteoporosis prevention and management, SIs exert multimodal effects. Their molecular structural homology with 17β-estradiol enables selective binding to estrogen receptor β (ERβ), conferring estrogen-like activity in bone tissue while eliciting no detectable stimulation in mammary or uterine tissues. Studies have demonstrated that SI intervention significantly elevates the serum levels of estradiol (E2), osteocalcin (BGP), and procollagen type I N-terminal propeptide (PINP) in ovariectomized (OVX) mice, concurrently reducing the concentration of C-terminal telopeptide of type I collagen (CTX-1) and restoring bone turnover biomarkers toward physiological ranges [48]. Supplementation with SIs markedly enriches the gut Verrucomicrobiota, with a pronounced increase in colonization by the genus Akkermansia. Notably, the keystone probiotic *Akkermansia muciniphila* strain Y5.1 can alleviate bone loss caused by estrogen deficiency. It exerts the protective effect through three complementary mechanisms: reinforcing intestinal barrier function, suppressing osteoclast differentiation from bone marrow monocytes/macrophages, and enhancing osteogenic differentiation of bone marrow mesenchymal stem cells. SI intervention was shown to significantly reduce the serum levels of triglycerides (TGs) and total cholesterol (CHO) in OVX mice, thus mitigating abnormal bone metabolism associated with lipid metabolism dysregulation [49]. And through suppression of the NF-κB signaling pathway, SIs reduce the production of pro-inflammatory cytokines TNF-α and IL-6, consequently attenuating systemic inflammation. Moreover, SIs, as an antioxidant, scavenge reactive oxygen species (ROS) and inhibit oxidative stress-induced osteoblast apoptosis [50].

#### 3.1.2. Yam Tuber Proteins

Yam tuber proteins, particularly bioactive peptides liberated through in vitro simulated gastrointestinal digestion, have exhibited multifaceted bioactivities. These include significant antioxidant capacity, genoprotective effects against DNA damage, angiotensin-converting enzyme (ACE) inhibitory activity, and growth suppression against specific pathogens such as *Escherichia coli* and *Salmonella* spp. [51]. Notably, aberrant activation of oxidative stress, inflammatory cascades, and the renin–angiotensin system (RAS) is a critical determinant of disrupted bone metabolic equilibrium and compromised skeletal tissue integrity [52,53,54]. Given their proven efficacy in providing antioxidant protection, exerting anti-inflammatory effects (indirectly via antimicrobial activity), and inhibiting angiotensin-converting enzyme (ACE), the multifunctional properties of yam protein derivatives suggest their potential therapeutic utility in modulating bone metabolism.

In vivo studies have demonstrated that yam proteins modulate skeletal homeostasis through intestinal immune microenvironment regulation. They significantly recalibrate local gut immunoglobulin levels (IgA, IgG, and IgM) and pro-inflammatory cytokines (e.g., TNF-α and IL-6), thereby mitigating immune injury induced by chemotherapy and other stress-inducing insults [51]. Yam proteins were also shown to remodel the architecture of gut microbiota, evidenced by significantly elevated abundance of beneficial taxa (e.g., *Lactobacillus* genus) alongside suppressed proliferation of potentially pathogenic genera including Desulfovibrio and Helicobacter [55]. Collectively, these regulatory actions on intestinal immunity and microbiota play pivotal mechanisms through which yam proteins influence bone metabolism via the GBA. In vitro investigations have further demonstrated that bioactive constituents isolated from yam tubers (e.g., dioscorin and diosgenin) directly stimulate osteoblast activation, as evidenced by enhanced osteogenic differentiation capacity in the MC3T3-E1 pre-osteoblastic cell line [56].

In summary, yam proteins, via bioactive peptides derived from their digestion, possess multiple bioactivities and mediate potential osteoprotective effects through modulation of the gut immune–microbiota axis and the direct action on osseous cells.

### 3.2. Plant Polyphenolic Compounds

Beyond plant-derived proteins, polyphenolic phytochemicals constitute another pivotal class of GBA regulators, attracting sustained research interest due to their broad-spectrum bioactivities and well-defined metabolic pathways. These compounds exert multi-targeted modulatory effects through three primary mechanisms, remodeling gut microbiota architecture, fortifying intestinal barrier integrity, and regulating bone metabolism-associated signaling cascades (e.g., OPG/RANKL axis) respectively. This section will focus on representative polyphenols.

#### 3.2.1. Naringin

Naringin, predominantly present in citrus fruits, enhances intestinal barrier function by increasing ileal villus density and upregulating tight junction protein expression. Regarding bone metabolism, naringin suppresses osteoclastogenesis by downregulating the RANKL/OPG ratio and inhibiting the expression of nuclear factor of activated T-cells 1 (NFATc1), a master transcriptional regulator of osteoclast differentiation [57]. Animal studies showed that naringin intervention significantly increased bone mineral density (BMD) in OVX mice while enhancing trabecular microarchitecture density, with efficacy comparable to SI [58]. Notably, among isoflavones, daidzein (the major soy isoflavone) is the primary substrate metabolized by gut microbiota into equol, while genistein is not converted to equol.

#### 3.2.2. Curcumin

Curcumin, the primary bioactive constituent of *Curcuma longa*, mitigates intestinal inflammation by enhancing gut microbiota diversity, increasing beneficial taxa such as *Bifidobacterium* and *Akkermansia*, and reducing the Firmicutes/Bacteroidetes (F/B) ratio [59]. Beyond gut protection, curcumin exerts pleiotropic bone-protective effects: it suppresses osteoclast activation and oxidative stress via induction of the Nrf2/HO-1 antioxidant pathway, while simultaneously potentiating osteoblast differentiation and bone formation through stimulation of the Wnt/β-catenin signaling cascade [60]. In vivo, curcumin improves bone mineral density and trabecular microarchitecture in osteoporotic models, partly by restoring gut barrier integrity and lowering systemic inflammation [61].

#### 3.2.3. Resveratrol

Resveratrol is a natural polyphenol widely distributed in Vitis vinifera, Arachis hypogaea and other edible plants [62]. It exerts prominent bone-protective effects via multiple regulatory mechanisms. Resveratrol regulates the balance of Th17/Treg cells and inhibits the secretion of pro-inflammatory cytokine IL-17, which effectively blocks IL-17-mediated excessive bone resorption [63]. In addition, resveratrol acts as a potent activator of sirtuin 1 (Sirt1) deacetylase. The activation of Sirt1 can delay the senescence of osteoblasts, prolong the cell lifespan, and further promote osteogenic differentiation and bone matrix mineralization [64]. Meanwhile, it can also alleviate oxidative stress and systemic inflammation, playing a synergistic role in maintaining bone homeostasis.

### 3.3. Plant-Derived Amino Acids and Peptides

As the third pivotal class of plant-derived GBA regulators, plant-sourced amino acids and peptides directly modulate bone remodeling processes through metabolic transformation and signal transduction. Whether directly absorbed intestinally or microbially metabolized into bioactive molecules, these components coordinately regulate osteoblast differentiation, osteoclast activity, and bone matrix mineralization. This section focuses on various core constituents.

#### 3.3.1. L-Arginine/Citrulline Metabolic Pathway

The gut microbiota family Lachnospiraceae modulates skeletal adaptation to mechanical loading through conversion of L-citrulline to L-arginine. Subsequent L-arginine metabolism in osteocytes gives rise to nitric oxide (NO) production, thereby initiating an NO–calcium positive feedback loop that augments the mechanosensitivity of osteocytes. Consequently, L-citrulline supplementation was shown to significantly enhance bone mechanical responsiveness and increase bone strength in normal, aged, and ovariectomized (OVX) murine models [65].

#### 3.3.2. Branched-Chain Amino Acids

Plant-derived proteins from legumes or whole grains, for example, contain high concentrations of branched-chain amino acids (BCAAs), including leucine, isoleucine, and valine. BCAAs promote osteoblast proliferation through mTOR complex 1 (mTORC1) signaling activation while also inhibiting osteoclast differentiation. BCAA intervention significantly increased the trabecular number and thickness in murine models, thereby ameliorating bone microarchitecture [66].

#### 3.3.3. Bioactive Peptides

Bioactive peptides derived from plant protein hydrolysis (e.g., soybean-derived lunasin) modulate gut microbiota composition by elevating Bifidobacterium and *Lactobacillus* abundance while reducing intestinal pH to enhance calcium solubilization and absorption. These peptides also exhibit antioxidant and anti-inflammatory activities, mitigating oxidative stress-induced damage to osteoblasts [67].

**Table 1:** Mechanisms of bioactive compounds regulating bone metabolism via the gut–bone axis.

Class	Active Compound	Key Gut Effects	Skeletal Effects and Clinical Implications
Plant proteins	Soy protein	Remodels the gut microbiota by enriching beneficial taxa, particularly increasing the abundance of *Akkermansia muciniphila* [68] Promotes the production of short-chain fatty acids (SCFAs) [69] Attenuates firmicutes/bacteroidetes (F/B) ratio [70]	Modulates bone homeostasis via dual anabolic–catabolic regulation [71]
		Performs immune modulation through tight junction protein upregulation (e.g., ZO-1, occludin) [72] Inhibits the expression and secretion of pro-inflammatory cytokines (e.g., TNF-α, IL-6) [73]	
	Yam tuber protein	Enhances microbial α-diversity and enriches beneficial taxa [74]	Inhibits osteoclast activity to improve trabecular connectivity and restore bone microarchitecture [56,75]
		Suppresses bone marrow-derived pro-inflammatory cytokines (e.g., TNF-α, IL-1β) [75]	
Plant polyphenols	Naringin	Promotes enrichment of beneficial bacteria [76] Promotes butyrate production [77]	Increases the bone formation rate [78] Reduces TRAP activity [79] Elevates trabecular number [80]
		Barrier function and immune regulation: Enhances intestinal barrier integrity (upregulating the expression of junction protein ZO-1) [81] Reduces the RANKL/OPG ratio [82]	
	Curcumin	Decreases the levels of pro-inflammatory mediators [83]	Increases bone mineral density [84]
		Increases the abundance of *Lactobacillus* spp. [85] Reinforces intestinal barrier integrity [86] Promotes ROS scavenging [87] Elevates SOD activity [87,88]	Prevents GC-induced bone loss [88]
	Resveratrol	Enhances the abundance of probiotic bacteria [89]	Suppresses osteoclast activity and exerts anti-inflammatory effects [90] Promotes osteogenic activity and suppresses osteoclastic bone resorption [91]
		Elevates cellular antioxidant capacity and anti-inflammatory activity [92] Inhibits NF-κB signaling, thereby attenuating inflammatory responses [93]	
Amino acids and peptides	L-Citrulline precursors	Enriches beneficial microbiota and exerts anti-inflammatory effects [94]	Restores trabecular microarchitecture [95] Promotes cortical osteogenesis and reduces cortical bone resorption [96]
		Maintains gut barrier integrity [97]	
		Improves intestinal function and mediates immunomodulation [98]	
	Branched-chain AAs (BCAAs)	Potentiates osteogenesis [99] Augments bone mineral density and mechanical strength [100]	Enhances bone mechanical strength [101] Modulates bone metabolism in the elderly [102]
		Elevates the abundance of probiotic genera [103] Promotes osteogenic differentiation [104]	
	Bioactive peptides	Inhibits osteoclastic bone resorption [105]	Promotes neo-osteogenesis at the fracture site [106] Accelerates stage-specific fracture healing [107]
		Enhances enteric anti-infective capacity [108] Remodels the gut microbiota and induces the expression of antimicrobial peptides [109]	
Probiotics	*BL*-11	Increases the abundance of probiotic genera [110] Potentiates bone mineralization and mechanical strength [111]	Potentiates longitudinal bone growth in pediatric populations [112]
		Inhibits osteoclastic resorption [113] Suppresses osteoclastogenesis [114]	
	*L. rhamnosus* GG (LGG)	Enhances gut barrier function and ameliorates inflammation [115,116]	Optimizes bone material properties [117] Attenuates fracture risk by augmenting bone structure [118]
		Exerts anti-inflammatory effects [119]	
Prebiotics/ symbiotic	Fructooligo- saccharides (FOSs)	Elevates the abundance of beneficial taxa [120]	Enhances calcium bioavailability, thereby augmenting bone mineral density [121]
		Exerts anti-inflammatory effects, enhances intestinal barrier function, and mediates immunomodulation [122]	

## 4. GBA-Targeted Intervention Strategies

### 4.1. Probiotics and Prebiotics

**Figure 2:**
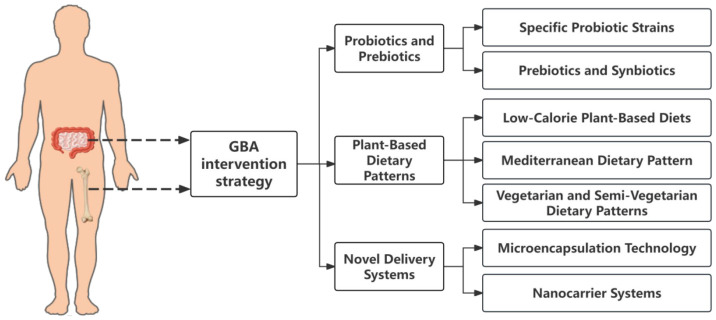
Schematic of GBA-based intervention strategy.

Microbiota-targeted therapeutic strategies grounded in GBA theory demonstrate significant clinical translational potential (Figure 2 and Table 1).

#### 4.1.1. Specific Probiotic Strains

*Bifidobacterium animalis* subsp. *lactis BL*-11 (*BL*-11), a mammalian-derived probiotic strain, enhances growth hormone secretion via high-yield γ-aminobutyric acid (GABA) production, thereby increasing insulin-like growth factor-1 (IGF-1) synthesis. It also potentiates osteocalcin activity through stimulation of menaquinone-7 (vitamin K_2_) biosynthesis [123]. *Lactobacillus rhamnosus GG* (LGG), a probiotic strain with an established long-term safety profile, demonstrates significant osteoprotective efficacy when delivered via sodium hyaluronate-based spherical microparticles (SMASMs). This delivery system was shown to enhance intestinal colonization capacity, increasing LGG viability by 84% and prolonging gut retention duration 2.8-fold. Consequently, LGG-SMASM treatment promotes short-chain fatty acid (SCFA) metabolism while suppressing expression of osteoclastogenic master regulators TRAF6 and NFATc1, ultimately increasing trabecular bone volume by 30.6% in osteoporotic murine models [124].

#### 4.1.2. Prebiotics and Synbiotics

Fructooligosaccharides (FOSs) and galactooligosaccharides (GOSs) are indigestible dietary constituents classified as prebiotics. They can selectively stimulate the proliferation and metabolic activity of beneficial gut microbiota (e.g., Bifidobacterium and *Lactobacillus* genera), thereby modulating intestinal microecological homeostasis. Their osteo-promotive mechanisms entail enhanced synthesis of microbiota-derived short-chain fatty acids (SCFAs), which subsequently increase mineral solubility and calcium absorption efficiency through intestinal lumen acidification while systemically modulating immune homeostasis [125].

Human milk oligosaccharides (HMOs), quintessential natural prebiotics, demonstrate significant osteoprotective effects in malnourished animal models. Post-intervention observations reveal not only an increased bone volume fraction (BV/TV) and trabecular number but also enhanced intestinal barrier integrity and elevated calcium bioavailability. These outcomes collectively indicate that HMOs improve bone metabolism through the microbiota–gut–bone axis regulatory framework [126].

Synbiotics are probiotic–prebiotic complexes that exert synergistic effects. They are exemplified by dual-component soy isoflavone/oligosaccharide interventions that are significantly superior to singular treatments in enhancing Akkermansia colonization rates and bone mineral density (BMD) improvement [127].

### 4.2. Plant-Based Dietary Regimens

Specific plant-centric dietary regimens modulate GBA functionality via the synergistic interplay of their bioactive constituents, encompassing three prototypical models.

#### 4.2.1. Low-Calorie Plant-Based Diet

Plant-derived dietary regimens that mimic fasting (characterized by low caloric/protein intake and high unsaturated fatty acid content) partially reverse intestinal damage in inflammatory bowel disease (IBD) murine models [128]. Beyond this fasting–mimicking diet (FMD), several representative plant-based regimens have shown gut-protective and bone-beneficial effects, including the Mediterranean diet, vegan diet, and whole-food plant-based diet (WFPBD). This intervention increases intestinal stem cell populations by 28% (*p* < 0.05), diminishes pro-inflammatory cytokine release, and indirectly ameliorates the bone metabolic microenvironment [129]. The FMD, in particular, is a 5-day plant-rich, low-calorie protocol that modulates gut microbiota, enhances intestinal barrier function, and reduces systemic inflammation—all of which support bone homeostasis [130]. Similarly, the Mediterranean diet, abundant in fruits, vegetables, whole grains, and olive oil, enriches short-chain fatty acids (SCFAs) and balances the Firmicutes/Bacteroidetes ratio, thereby inhibiting osteoclastogenesis [131]. These dietary regimens are enriched with phytochemicals that confer anti-inflammatory and antioxidant effects through modulation of gut microbial diversity.

#### 4.2.2. Mediterranean Diet

This plant-centric regimen, characterized by whole grains, fruits, vegetables, legumes, and extra virgin olive oil, provides abundant polyphenols, dietary fiber, and ω-3 polyunsaturated fatty acids (PUFAs). The diet maintains osseous metabolic equilibrium through three synergistic mechanisms, enriching SCFA-producing taxa, attenuating intestinal permeability, and reducing systemic inflammation respectively [132]. Epidemiological evidence reveals that sustained adherence to a Mediterranean diet significantly attenuates BMD loss rates and reduces hip fracture risk in postmenopausal women [133].

#### 4.2.3. Vegetarian and Semi-Vegetarian Diets

Well-planned vegetarian regimens provide sufficient plant-derived proteins, minerals, and diverse phytochemicals. Research studies have demonstrated comparable BMD between lacto–ovo vegetarians and omnivores, whereas vegans necessitate focused calcium, vitamin D, and vitamin B12 supplementation to mitigate nutritional deficits. Furthermore, the abundant potassium and magnesium inherent in these diets maintain the systemic acid-base equilibrium, thereby attenuating bone calcium resorption [134].

### 4.3. Novel Delivery Systems

In addressing the bioavailability challenges of plant-derived active compounds, the development of innovative delivery platforms has emerged as a pivotal research focus.

#### 4.3.1. Microencapsulation Technology

Hyaluronic acid-coated spherical microparticles (SMASMs) enhance intestinal mucosal adhesion through thiol-disulfide exchange reactions, thereby increasing bacterial strain viability and colonization efficiency [135]. In addition, liposomal encapsulation of soy isoflavones has been shown to significantly enhance bioavailability and potentiate osseous tissue targeting [50,136].

#### 4.3.2. Nanocarrier Systems

Silica nanoparticle-loaded naringin improved ovariectomized (OVX) murine bone microarchitecture by prolonging the duration of intestinal retention and potentiating Akkermansia colonization [137]. In addition, chitosan nanoparticles were shown to serve as efficient gene delivery vectors, such as enabling targeted shRNA delivery to silence RANK or TRAF6 genes and thereby inhibit osteoclast differentiation [138].

## 5. Discussion

The gut–bone axis (GBA) framework provides a novel paradigm for osteoporosis prevention and treatment. Plant-derived bioactive compounds and probiotics demonstrate considerable therapeutic potential through multi-targeted modulation of intestinal microecology and skeletal homeostasis. Current evidence confirms that phytochemicals (soy isoflavones, naringin, curcumin, resveratrol, etc.) significantly improve bone mineral density via enrichment of beneficial intestinal taxa (e.g., Akkermansia), restoration of intestinal barrier integrity, and suppression of systemic inflammation. Precision interventions grounded in GBA theory, including specific probiotic strains (e.g., BL-11), plant-centric dietary regimens, and advanced biomaterial delivery systems, have demonstrated significant clinical potential. For instance, clinical trials have validated that soy isoflavones and genistein significantly improve bone mineral density and bone metabolism in postmenopausal women and children [10]. These pieces of clinical evidence strongly support the translational value of gut–bone axis targeted strategies. Nevertheless, critical knowledge gaps persist regarding dose–response relationships, strain-specific mechanisms of action, and long-term safety profiles, necessitating rigorous evaluation, particularly in hormone-sensitive populations. This review systematically summarizes the regulatory effects and underlying mechanisms of plant-derived bioactive components, probiotics and plant-based dietary patterns on bone metabolism from the perspective of the gut–bone axis. We combed a large number of recent in vitro experiments, animal studies and clinical trials, clarified the core signaling pathways and key microbial taxa involved in the regulation process, and sorted out the latest progress of targeted delivery technologies, which can provide a comprehensive reference for subsequent basic research and clinical transformation in this field.

However, several limitations still exist. Most relevant studies are still limited to animal models and in vitro cell experiments, and large-sample, long-term human clinical trials remain insufficient [139]. The bioavailability of most plant active compounds is low, and the optimal administration route, dosage and intervention cycle have not formed unified standards. Individual differences in gut microbiota lead to inconsistent intervention effects, and the precise regulatory network between complex microbial communities and bone metabolism has not been fully elucidated. In addition, this review does not cover regional dietary differences and population heterogeneity in detail, which needs to be supplemented in follow-up research.

In view of the existing deficiencies and research gaps, multiple research directions deserve priority exploration in the future. First, multi-omics technologies including metagenomics, metabolomics and transcriptomics should be comprehensively integrated to reveal the dynamic interaction network between gut microbiota, microbial metabolites and bone metabolism, and construct personalized precision intervention models based on individual microbiome characteristics. Second, standardized experimental designs are required to clarify the dose–effect relationship of plant bioactive compounds and the functional differences among different probiotic strains, so as to determine the optimal intervention dosage and matching strains. Third, more long-term clinical trials should be carried out to evaluate the safety and efficacy of gut–bone axis targeted therapies in special populations such as postmenopausal women, elderly people and patients with chronic diseases. Fourth, combined with nanotechnology and biomaterials, novel delivery systems need to be developed to improve the solubility, stability and bioavailability of plant active ingredients, so as to enhance the practical application value of related interventions. With the continuous deepening of basic research and the innovation of delivery technologies, microecological regulation strategies based on plant-derived components will become a safe and effective auxiliary means for the prevention and treatment of osteoporosis and other metabolic bone diseases.

## 6. Conclusions

Osteoporosis is a common chronic metabolic bone disease closely associated with intestinal microecological disorders. The gut–bone axis builds an important bridge connecting intestinal health and skeletal homeostasis. A variety of natural plant-derived bioactive substances, represented by soy isoflavones, naringin, curcumin and resveratrol, together with functional probiotics and rational plant-based low-calorie diets, can jointly regulate the composition of gut microbiota, maintain intestinal barrier integrity, reduce the systemic inflammatory response and oxidative stress, and further regulate osteoblast and osteoclast activities through multiple signaling pathways, thus exerting favorable bone-protective effects. At present, gut–bone axis targeted interventions have shown good application prospects in preclinical and partial clinical studies. Although there are still challenges, such as unclear molecular mechanisms, insufficient clinical evidence and unstandardized dosage regimens, the continuous progress of multi-omics technology and novel delivery materials will effectively promote the development of this field. In the future, gut microbiota-based microecological intervention is expected to become a new safe strategy for the prevention and clinical adjuvant treatment of osteoporosis.

## Data Availability

No new data were created or analyzed in this study.
